# Anger is eliminated with the disposal of a paper written because of provocation

**DOI:** 10.1038/s41598-024-57916-z

**Published:** 2024-04-09

**Authors:** Yuta Kanaya, Nobuyuki Kawai

**Affiliations:** 1 Present Address: Department of Cognitive and Psychological Sciences, Nagoya University, Nagoya, 464-8601 Japan; 2 Academy of Emerging Science, Chubu University, Kasugai City, 487-8501 Japan

**Keywords:** Anger, Management, Suppression, Disposal, Rumination, Human behaviour, Psychology

## Abstract

Anger suppression is important in our daily life, as its failure can sometimes lead to the breaking down of relationships in families. Thus, effective strategies to suppress or neutralise anger have been examined. This study shows that physical disposal of a piece of paper containing one’s written thoughts on the cause of a provocative event neutralises anger, while holding the paper did not. In this study, participants wrote brief opinions about social problems and received a handwritten, insulting comment consisting of low evaluations about their composition from a confederate. Then, the participants wrote the cause and their thoughts about the provocative event. Half of the participants (disposal group) disposed of the paper in the trash can (Experiment 1) or in the shredder (Experiment 2), while the other half (retention group) kept it in a file on the desk. All the participants showed an increased subjective rating of anger after receiving the insulting feedback. However, the subjective anger for the disposal group decreased as low as the baseline period, while that of the retention group was still higher than that in the baseline period in both experiments. We propose this method as a powerful and simple way to eliminate anger.

## Experiment 1

### Introduction

The need to control anger has been of importance for a long time in human societies, as inferred by a philosopher in Imperium Romanum who had already explored how to cease being angry^1^. However, it can still be challenging to suppress anger effectively. Frequent, unregulated anger often leads to violence towards children^2^, which has become an increasingly prevalent issue. One study found that the global estimate for children experiencing any form of violence (physical, sexual, emotional, or a combination) in the past year is one billion children aged 2–17 years^3^. The number of child abuse cases in Japan has reportedly doubled in the past decade^4^. Children learn about appropriate emotional expression and behaviour from their parents^5^, and children who have been maltreated may lack the opportunity to learn how to regulate anger. Consequently, these maltreated children may have difficulty controlling their own anger^6^, recognising anger in others^7^, and tend to exhibit externalizing behaviour problems^8^. These studies suggest that parental anger regulation issues negatively affect children’s emotional competence. Therefore, an effective way of reducing anger has been examined throughout the years^9^.

However, simply attempting to suppress anger is usually not effective^10^. Both cognitive reappraisal and distraction (i.e., thinking about something other than provocative comments) could reduce anger; however, distraction could suppress anger only for a transient period of time^11^. Cognitive reappraisal refers to the reinterpretation or modification of the meaning of an unpleasant situation. Although reappraisal is considered as an effective way to reduce anger^12^, it requires greater cognitive effort^13,14^. Therefore, reappraisal under stressful situations which require cognitive load was not found to be effective in reducing anger as compared to non-stressful situations^15^. Self-distancing, which may be responsible for the anger-reducing effect of reappraisal^12^ is also considered as an effective way to reduce anger. Nevertheless, self-distancing or reflection on one’s provocation from a distance is often not feasible, especially in the heat of the moment^13^.

Failure to reduce anger can lead an individual to think about a provocative event repeatedly. Such ruminations are often produced in a self-immersed, experiential manner^16^. Self-immersed experiential rumination can lead to reliving past provocative events^17^, thus maintaining or even increasing subjective anger and vascular responses^18^.

However, among the types of ruminations, writing down a provocation event does not always maintain or increase anger; instead, anger is suppressed depending on the way of writing. For instance, anger was suppressed when participants wrote down the anger-inducing event in a detached, informational, ‘cool’ manner. However, their anger was not suppressed (and was maintained or even increased) when they failed to write down the event in an analytical manner, and wrote it down in a ‘hot’ (emotional) manner^12^. Somewhat relevant here is the expressive writing technique^19^, which is frequently used in emotion-focused psychotherapy treatment^20^. It is believed to be effective in suppressing anger in clinical settings. However, only one experimental study using this technique has been conducted, wherein it was found that there was a significant likelihood of reduced anger when sentences about the emotion were written in the past tense^21^. These studies suggest that anger may be successfully suppressed if individuals are able to separate their internal experience of provocative events from their sense of self^22^. Healy et al.^23^ reported that negative self-referential statements (‘my life is pointless’), when presented in a defused format (‘I am having a thought that my life is pointless’), could decrease the emotional discomfort related to that statement.

These previous studies emphasised the cognitive processes (such as goals or valuations) that occur almost entirely inside individuals’ heads^24^. However, if we look at the literature more broadly, studies on emotion regulation (a situated cognitive approach) have demonstrated successful emotion control through dynamic interplay between the person and the situation^24,25^. From this situated cognition perspective, people perceive their environment in terms of the possibilities for the kinds of actions that they would pursue. These functional features of the environment (affordances) do not solely exist inside an individual’s mind but instead have a physical reality that exists in the individual’s relationship with the environment. For instance, people frequently use physical substances to modify their moods. People may take a hot shower when they feel lonely^26,27^ or hold a teddy bear when they feel afraid^28^. Such access to physical objects can significantly modify individuals’ ability to manage their emotions.

In this study, we developed a new anger reduction strategy inspired by the situated cognition approach to emotion regulation^24^. Relevant to this approach, the notion of a grounded procedure of separation^29^ also assumes that mental representations and functions are grounded in one’s own experiences and interactions with physical reality. For instance, if people want to take revenge through permanent removal (e.g. hatred for ex), they may destroy a related entity such that it is no longer recognisable (burn, melt, or tear related). In a related study, Briñol et al.^30^ reported that writing down negative thoughts about a Mediterranean diet on a piece of paper and disposing of the paper in a trash can result in lower negative (more positive) evaluations of the diet, compared to a group that kept the paper in a booklet. These attitude changes may derive from the cognitive fusion that people often fuse with physical objects, such as jewellery, cars, and family heirlooms^31^. Such fused objects are valued more and are less likely to be abandoned because doing so means losing a part of themselves^32,33^. Specifically, throwing an object associated with negative emotions (anger) may result in losing the negative emotions (anger). However, to the best of our knowledge, no study has tested whether the disposal of anger-written paper can reduce or even eliminate anger.

Previous studies from a situated cognitive approach to anger management have changed the external environment of the individual in anger. Tool (object) use has received scant attention in these situated cognition approaches to anger management, except for a few studies, such as hitting a punching bag^34^ and playing a video game^35^. This study examined a method in which the disposal of a paper (object) on which participants wrote down their descriptions or thoughts about a provocative event could neutralise anger. Participants threw the anger-written paper into a trash box in Experiment 1, and put the paper into a shredder in Experiment 2. If the action of disposal is crucial to modifying emotions, anger would be reduced only in participants in Experiment 1 but not in Experiment 2, as predicted by the grounded separation procedure^29^. Nevertheless, if anger was modified by the meaning of disposal, the subjective ratings of anger would be eliminated in both experiments. The disposal of the paper with the written descriptions would remove the psychological existence of anger for the provoked participants along with the disposal of paper by the dynamic interactions with the object^24^. This simple method of eliminating anger could potentially contribute to effective parental anger management toward their children.

### Materials and method

#### Participants

A total of 57 students (women = 21, mean age = 21.11, *SD* = 1.05) from a local university participated in this experiment. The data from seven participants were excluded from the final analysis because they correctly guessed the purpose of the experiment and they did not express induced anger by insult (subjective ratings of anger were lower or the same compared to those of the baseline), as was the case in a previous study^36^. Our final analysis included 50 participants (women = 16, mean age = 21.10, *SD* = 1.08). A sample size of 50 participants was determined by G*Power 3.1.9.4^37^ using the a priori procedure for repeated measures ANOVA, within (periods)—between (disposal and retention) interaction with the parameters of 95% power, an expected effect size of 0.25 (defined as a medium effect by Cohen^38^), alpha level of 0.05, a within-subjects measurement correlation of 0.5, and a nonsphericity correction ε of 1. The calculation suggested a sample size of 22 participants in each group. Based on these analyses, we concluded that the sample size was appropriate for this study.

#### Materials

Angry feelings were assessed with five adjective items: angry, bothered, annoyed, hostile, and irritated. These adjectives were previously used as measures of self-reported anger^39^. In this study, each response scale ranged from 1 (not at all) to 6 (extremely). As was the case in a previous study on anger^40^, scores on these five adjectives were averaged to form an anger experience composite, which was the score used in the analysis (Cronbach’s α = 0.90). We also used Positive and Negative Affect Schedule (PANAS) as a subjective scale to assess mainly negative feelings^38^. We used the Japanese version of the 6-point PANAS scale^41^.

#### Procedure

In this experiment, participants' subjective emotional states were measured at three time points (baseline, post-provocation, and post-writing). The participants were told to write an essay on social problems (e.g., smoking in public) for which they would receive feedback from a doctoral student assessing the quality of the essay. They had seen the doctoral student before entering the experimental room. After the participants wrote the essay, they completed the PANAS and anger questionnaires for the baseline. The evaluation by the fictitious doctoral student was then provided to the participants. The evaluation included ratings of the essay on six characteristics using a 9-point scale (e.g. for intelligence, 1 = unintelligent, 9 = intelligent). All participants were given the following ratings: intelligence = 3, interest = 3, friendliness = 2, logic = 3, respectability = 4, and rationality = 3. Each essay was also provided with the following comment: ‘I cannot believe an educated person would think like this. I hope this person learns something while at the university’^40,42^. All of these manipulations were successfully used in our previous study^40^. The participants were required to read the feedback ratings and comments silently for two minutes. Then, they filled out the subjective emotional questionnaires (PANAS and anger adjectives) for the post-provocation period.

Then, the participants were asked to write every thought of them on receiving the feedback and were given three minutes for this. The instruction was ‘Think about the event from your own perspective. Concentrate especially on the things that originally triggered the emotions and your reactions’. We added guide questions (‘Why were you feeling this way?’, ‘What made you feel this way?’) to induce analytical rumination. To allow the participants to write about their honest feelings, they were informed that the written paper would not be seen by anyone, including the experimenter. After writing, the participants were asked to review the sentences carefully for 30 s. For the retention group, the paper was turned over, put in a clear plastic folder, and placed on the right side of the desk. The participants in the disposal group rolled up the paper into a crumpled ball, stood up, threw the paper into the trash can held by the experimenter, and sat back in the chair. Finally, both groups of participants filled out the subjective emotional questionnaires (anger adjectives and PANAS) for the post-writing period. At the end of the experiment, all participants were debriefed and informed of the truth. They were also assured that the evaluations of their essays had been prepared in advance.

### Data analyses

Angry feelings were analysed using a 2 (group: disposal or retention) × 3 (period: at baseline, post-provocation, and post-writing) ANOVA. All significance levels were set at *p* < 0.05. We used the Greenhouse–Geisser correction when Mauchly’s test of sphericity was violated. When the interaction was significant, multiple comparisons using the Bonferroni correction method were used to assess the differences.

We also report Bayes factors (BFs) from the Bayesian repeated measures ANOVA in *JASP*^43^. For BFs, BF_10_ values reflect the probability of an alternative relative to the null hypothesis. BFs greater than 3 indicate support for the hypotheses. A BF favouring the alternative over the null hypothesis (BF_10_) offers strong evidence for the alternative hypothesis when it is over 10. Values less than 0.33 indicate support for the null hypothesis, and values between 0.33 and 3 indicate data insensitivity. We also reported 95% confidence intervals.

We aimed to examine (1) whether angry feelings resumed in the disposal group, and (2) whether angry feelings were different between the groups after the disposal or retention treatments. Our main interest was angry feelings, while we also verified PANAS scores using a 2 (group: disposal or retention) × 3 (period: at baseline, post-provocation, and post-writing) ANOVA.

#### Ethics statement

All participants were paid for their participation and had provided written informed consent in accordance with the procedures before participation. The study was approved by the Ethics Committee of the Department of Cognitive and Psychological Sciences at Nagoya University (201104-C-02–02). All methods were carried out in accordance with the ethical guidelines of the Declaration of Helsinki. All participants provided their written and informed consent prior to starting the study.

### Results

#### Anger experience

**Figure 1:**
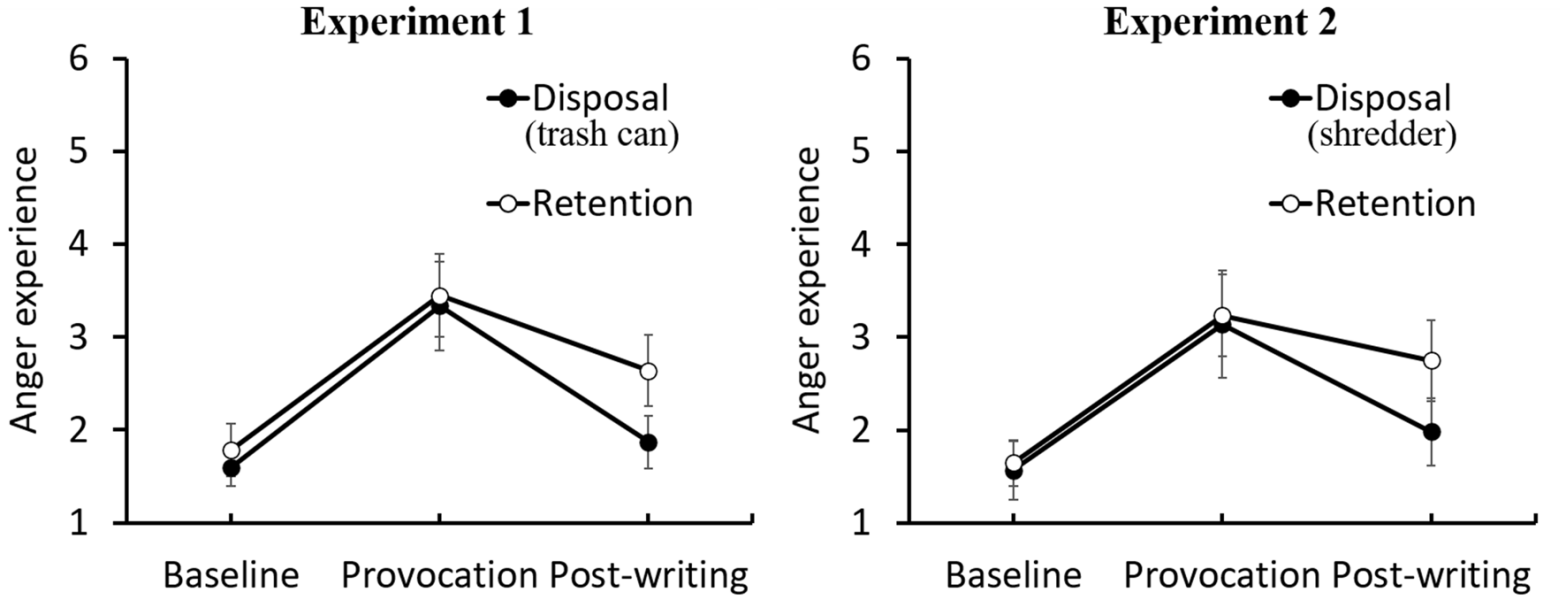
Self-reported anger during Experiment 1 (left) and Experiment 2 (right). Significant differences emerged at the end of time due to experimental manipulations. Possible values for anger range from 1 to 6. Each vertical line illustrates the 95% confidence intervals for each group.

The left panel of Fig. 1 shows mean subjective ratings of anger for disposal and retention groups at three time points (baseline, post-provocation, and post-writing). Subjective ratings of anger of both groups increased at the post-provocation (*M*_*disposal*_ = 3.34, *SD* = 1.20, 95% CI [2.86, 3.82]; *M*_*retention*_ = 3.45, *SD* = 1.11, 95% CI [3.00, 3.89]) from the baseline (*M*_*disposal*_ = 1.59, *SD* = 0.50, 95% CI [1.39, 1.79]; *M*_*retention*_ = 1.78, *SD* = 0.71, 95% CI [1.50, 2.07]). Subjective ratings at the post-writing decreased from the post-provocation, however those of retention group were still higher than the baseline (*M*_*retention*_ = 2.64, *SD* = 0.95, 95% CI [2.26, 3.02]), while those of disposal group eliminated at the same level of the baseline (*M*_*disposal*_ = 1.87, *SD* = 0.71, 95% CI [1.59, 2.16]). A 2 (group: disposal or retention) × 3 (period: at baseline, post-provocation, and post-writing) mixed model analysis of variance (ANOVA) revealed a significant main effect of period [*F* (2, 96) = 73.36, *p* < 0.001, partial *η*^2^ = 0.60, BF_10_ > 100], while a main effect of group was not significant [*F* (1, 48) = 3.21, *p* > 0.05, partial *η*^*2*^ = 0.06, BF_10_ = 0.66]. The interaction between group and period was significant [*F* (2, 96) = 3.12, *p* < 0.05, partial *η*^*2*^ = 0.06, BF_10_ = 1.17]. Multiple comparisons with the Bonferroni method revealed that the subjective anger was significantly higher at the post-provocation than those at the baseline (*p* < 0.05), indicating that a provocative manipulation was exerted. Subjective ratings of anger post-writing decreased significantly, compared to post-provocation (*p* < 0.05). Importantly, however, subjective ratings of retention group at the post-writing period were still significantly higher than those of the baseline period (*p* < 0.05), whereas those of disposal group at the post-writing period eliminated to levels of the baseline period (*p* > 0.05). Subjective ratings of disposal group at the post-writing period were significantly lower than those of retention group (*p* < 0.01).

#### Negative and positive affect

The negative affect subscale of the PANAS at post-provocation (*M*_*disposal*_ = 3.10, *SD* = 1.00, 95% CI [2.70, 3.49]; *M*_*retention*_ = 3.06, *SD* = 1.03, 95% CI [2.64, 3.47]) was higher than at baseline (*M*_*disposal*_ = 2.45, *SD* = 0.66, 95% CI [2.18, 2.71]; *M*_*retention*_ = 2.50, *SD* = 0.84, 95% CI [2.16, 2.83]) and post-writing (*M*_*disposal*_ = 2.06, *SD* = 0.65, 95% CI [1.80, 2.32]; *M*_*retention*_ = 2.39, *SD* = 0.88, 95% CI [2.04, 2.73]). The 95% CIs of the disposal group overlapped a little bit between post-provocation [2.70, 3.49] and baseline periods [2.18, 2.71], and those of the retention group overlapped between both the post-provocation [2.64, 3.47] and baseline [2.16, 2.83]. The 95% CIs for the post-writing means partially overlapped between the groups. A 2 (group) × 3 (period) mixed ANOVA revealed a significant main effect of period [*F* (2, 96) = 28.64, *p* < 0.001, partial *η*^*2*^ = 0.37, BF_10_ > 100]. However, the main effect of group [*F* (1, 48) = 0.29, *p* > 0.05, partial *η*^*2*^ = 0.01, BF_10_ = 0.32] and the interaction between group and period were not significant [*F* (2, 96) = 1.35, *p* > 0.05, partial *η*^*2*^ = 0.03, BF_10_ = 0.31]. Multiple comparisons with the Bonferroni method revealed that the subjective negative affect post-provocation was significantly higher than at baseline and post-writing (*ps* < 0.05).

The PANAS positive affect subscale showed little variation at three periods (*M*_*disposal*_ = 2.33, *SD* = 0.80, 95% CI [2.01, 2.65]; *M*_*retention*_ = 2.32, *SD* = 0.75, 95% CI [2.01, 2.62]), post-provocation (*M*_*disposal*_ = 2.44, *SD* = 0.76, 95% CI [2.13, 2.75]; *M*_*retention*_ = 2.42, *SD* = 0.89, 95% CI [2.06, 2.78]), and post-writing (*M*_*disposal*_ = 2.38, *SD* = 0.87, 95% CI [2.03, 2.73]; *M*_*retention*_ = 2.27, *SD* = 0.83, 95% CI [1.93, 2.60]). A 2 × 3 mixed ANOVA revealed that neither main effects nor interaction was significant (*Fs* < 0.90, *ps* > 0.41, BF_10_s < 0.14).

### Discussion

This study examined whether writing about the provocative event and disposing of the paper into a trash can would suppress anger. The provocation treatments evoked anger in both the groups similarly. Nevertheless, the retention group still showed significantly higher anger compared to levels at the baseline period, while the disposal group completely eliminated their anger after the disposal of the anger-written paper. These results suggest that the disposal of the paper containing ruminated anger into the trash can neutralise anger. Our interpretation is that the act of throwing the paper with ruminated anger into the trash can produces a feeling similar to the psychological existence (anger) being discarded, leading to anger elimination, since the psychological entity (anger) was disposed along with the physical object (anger-written paper).

One may argue that it was not the disposal itself but the physical distance played a critical role in reducing anger. Since the paper was distanced from participants in the disposal group, whereas the paper in the retention group was located by them. Nevertheless, Zhang et al.^44^ showed that engaging in an avoidance action rather than creating physical distance was critical for reversing the perceived effect of negative thoughts. In their study (Experiment 5), participants in avoidance action conditions either threw the ball to the opposite corner of the room (creating physical distance between themselves and the ball), or pretended to throw the ball (creating no distance between themselves and the ball). Participants in the no-avoidance action condition either carried the ball to the opposite corner of the room and left it there (creating physical distance between the self and the ball without involving a throwing action) or held the ball in their non-dominant hand (creating no distance). Participants in both avoidance action conditions reversed the negative thoughts, while participants in both no-avoidance conditions did not. Avoidance actions were crucial in their study. Therefore, the physical distance would not contribute to reduce anger in this study. However, disposal action might be the key to neutralising anger in this study. Nevertheless, we assume that the meaning (i.e. interpretation) of disposal is more important than the action itself. Other studies have also suggested that the meaning of an action is critical for determining its impact, not the action itself^30,45^. This study could not exclude throwing action's potential contribution to neutralising anger. Thus, we conducted another experiment to exclude the potential contribution of the throwing action as much as possible, confirm the effectiveness of the disposal method, and explore the variation in this method.

## Experiment 2

### Introduction

Experiment 1 indicated that the disposal of a piece of paper containing the description of an anger-inducing experience into the trash can neutralise anger. However, it was unclear what aspect of the paper’s disposal neutralised anger. Although we interpreted the meaning of the action as critical to neutralising anger, the physical distance between the participant and the paper or the action itself (i.e. embodied cognition) might have played a critical role. We set up the second experiment: (1) to replicate the results of Experiment 1; (2) to exclude the embodied explanation as much as possible; and (3) to explore another version of the disposal method using a shredder on the desk. In this experiment, we asked participants to put the paper containing anger into the shredder instead of throwing it into the trash can which was kept at some distance from the participants. We also made a small change to the retention group. Participants of retention group put the paper into a clear box on the desk, and the disposal group put the paper into the shredder. Thus, the distance between the participants and the paper and the type of action were matched between the two groups. If the sensorimotor experience of throwing the paper was critical to neutralise anger, we would not be able to replicate the results of Experiment 1. Nevertheless, if the meaning of the disposal of a physical entity plays a critical role in reducing anger, we anticipated obtaining similar results. In line with our prediction, the attitude changed when the paper was transferred to a box labelled ‘trash can’, which indicated mentally discarding it, compared to a box labelled ‘safety box’^46^, suggesting that the perceived meaning of actions, and not the actions per se, influence attitude change. Hence, we designed a new study to confirm whether the perceived meaning of action eliminates anger. We predicted that putting the paper in a shredder would reduce negative emotions (anger), as compared to keeping the paper.

### Method

#### Participants

A total of 48 participants (women = 24, mean age = 26.81, *SD* = 9.42) were participated through worker dispatching company and a local university. There was no overlap between the participants of the two experiments. This sample size was determined using G*Power 3.1.9.4^37^ using the a priori procedure for repeated measures ANOVA, within (periods)–between (disposal and retention) interaction with the parameters of 95% power, an expected effect size of 0.25 (defined as a medium effect by Cohen^38^), alpha level of 0.05, a within-participants measurement correlation of 0.5, and a nonsphericity correction ε of 1. The calculation suggested a sample size of 22 participants in each group. Based on these analyses, we concluded that the sample size was appropriate for this study. As in Experiment 1, the data of two participants were excluded from the final analysis because they correctly guessed the purpose of the experiment and did not express anger by insult (subjective ratings of anger were lower or the same as those at the baseline). Our final analysis included 46 participants (women = 23, mean age = 26.39, *SD* = 9.14).

#### Materials

As in Experiment 1, angry feelings were assessed using five adjectives: angry, bothered, annoyed, hostile, and irritated. Responses ranged from 1 (not at all) to 6 (extremely). Scores on these five adjectives will be averaged to form an anger experience composite, which is the score used in the analyses. We also used the Japanese version of the 6-point PANAS scale as a subjective scale to assess mainly negative feelings^40,41^.

For the disposal group, a dustbin-type shredder (ACCO Brands Japan Corp, GSHA26MB) was used. This shredder (30 cm × 10 cm × 28 cm) cuts paper into pieces of 2 mm × 14 mm on putting the paper in from the top. The lower part of the shredder holds a transparent dustbin, so that the pieces of paper can be observed from the outside. For the retention group, a hand-made clear plastic box (23 cm × 5 cm × 30 cm) was used. Paper can be placed from the top, as with the shredder. Furthermore, as with the lower part of the shredder, the box is also transparent so that the paper in the box can be observed from the outside.

#### Procedure

**Figure 2:**
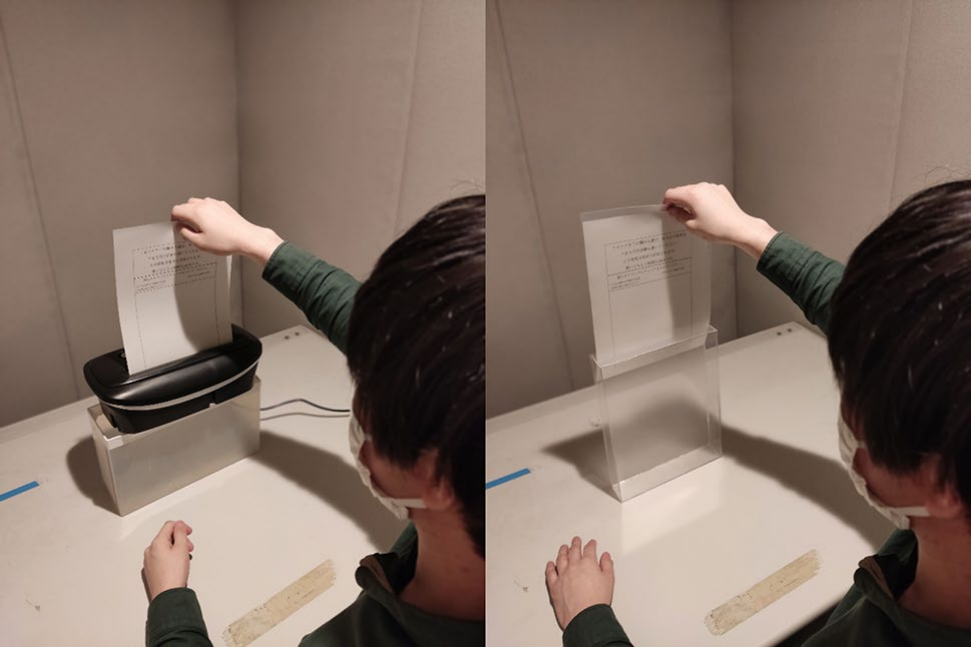
Pictures of experimental manipulations in Experiment 2. The disposal group (left) put the paper into the shredder, while the retention group (right) put the paper into the transparent box.

This experiment followed the same method used in Experiment 1 with slight changes. The words “while at university” were removed from the provocative comment (‘I cannot believe an educated person would think like this. I hope this person learns something while at university’^40,42^, because non-students participated in this study. The second change was the method of disposing or retaining the paper containing a description of the anger-inducing experience. After participants wrote down provocative events in an analytical manner, a transparent box or a transparent shredder bin was placed on the desk in front of them (Fig. 2), before they were asked to review the sentences carefully for 30 s. Then, participants were required to put the paper into the box, with the frontside of the paper facing them. Participants in the disposal group watched as the paper was cut in the shredder for five seconds. Participants in the retention group were required to enclose the paper in a clear file folder and place it in a transparent box showing their written sentences. Then, they observed the paper carefully for five seconds. Subsequently, the box was turned back to show the blank side of the paper. All participants rated their anger and provided responses to the PANAS after these treatments.

### Results

#### Anger experience

The right panel of Fig. 1 shows the mean subjective anger ratings for the disposal and retention groups at the three time points (baseline, post-provocation, and post-writing). This pattern of results is similar to that of Experiment 1. Subjective ratings of anger in both groups increased after provocation (*M*_*disposal*_ = 3.14, *SD* = 1.38, 95% CI [2.56, 3.72]; *M*_*retention*_ = 3.24, *SD* = 1.04, 95% CI [2.80, 3.67]) from baseline (*M*_*disposal*_ = 1.57, *SD* = 0.75, 95% CI [1.25, 1.88]; *M*_*retention*_ = 1.64, *SD* = 0.59, 95% CI [1.40, 1.89]). Subjective ratings at post-writing decreased from post-provocation. However, those of the retention group were still higher than those of the baseline (*M*_*retention*_ = 2.75, *SD* = 1.05, 95% CI [2.31, 3.19]), while those of the disposal group were eliminated at the same level as the baseline (*M*_*disposal*_ = 1.98, *SD* = 0.87, 95% CI [1.62, 2.35]). Only a small overlap (0.04) was observed in the 95% CI for the mean post-writing scores between the groups. A 2 (group: disposal or retention) × 3 (period: at baseline, post-provocation, and post-writing) mixed model ANOVA revealed a significant main effect of period [*F* (2, 88) = 56.93, *p* < 0.001, partial *η*^*2*^ = 0.56, BF_10_ > 100], while the main effect of group was not significant [*F* (1, 44) = 1.68, *p* > 0.05, partial *η*^*2*^ = 0.04, BF_10_ = 0.46]. The interaction between group and period was significant [*F* (2, 88) = 3.49, *p* < 0.05, partial *η*^*2*^ = 0.07, BF_10_ = 1.62]. Multiple comparisons with the Bonferroni method revealed that subjective anger was significantly higher at post-provocation than baseline (*p* < 0.05), indicating that provocative manipulation was exerted. Subjective ratings of anger at post-writing decreased significantly compared to post-provocation (*p* < 0.05). However, the subjective ratings of the retention group in the post-writing period were still maintained at the same level of anger as those of the post-provocation period (*p* > 0.05). Contrastingly, those of the disposal group in the post-writing period were significantly lower than those of the post-provocation period (*p* < 0.05).

Additionally, as was the result of Experiment1, the subjective ratings of the retention group in the post-writing period were significantly higher than those of the baseline period (*p* < 0.05). Those of the disposal group in the post-writing period were eliminated to the baseline period (*p* > 0.05). The subjective ratings of the disposal group in the post-writing period were significantly lower than those of the retention group (*p* < 0.05).

#### Negative and positive affect

The negative affect subscale of the PANAS at post-provocation (*M*_*disposal*_ = 3.34, *SD* = 1.09, 95% CI [2.88, 3.79]; *M*_*retention*_ = 3.35, *SD* = 0.89, 95% CI [2.98, 3.73]) was higher than at baseline (*M*_*disposal*_ = 2.60, *SD* = 0.78, 95% CI [2.27, 2.93]; *M*_*retention*_ = 2.73, *SD* = 0.92, 95% CI [2.34, 3.11]) and post-writing (*M*_*disposal*_ = 2.45, *SD* = 0.96, 95% CI [2.05, 2.85]; *M*_*retention*_ = 2.57, *SD* = 0.87, 95% CI [2.20, 2.93]). The 95% CIs of the disposal group overlapped a little bit between post-provocation [2.88, 3.79] and baseline periods [2.27, 2.93], and those of the retention group overlapped between both the post-provocation [2.98, 3.73] and baseline [2.34, 3.11]. A 2 (group) × 3 (period) mixed ANOVA revealed a significant main effect of period [*F* (2, 88) = 20.19, *p* < 0.01, partial *η*^*2*^ = 0.68, BF_10_ > 100]. However, the main effect of the group [*F* (1, 44) = 0.15, *p* > 0.05, partial *η*^*2*^ = 0.06, BF_10_ = 0.33] and the interaction between group and period were not significant [*F* (2, 88) = 1.35, *p* > 0.05, partial *η*^*2*^ = 0.05, BF_10_ = 0.13]. Multiple comparisons with the Bonferroni method revealed that the subjective negative affect post-provocation was significantly higher than at baseline and post-writing (*ps* < 0.05).

The positive affect subscale of the PANAS showed little variation at the three-time points (*M*_*disposal*_ = 2.88, *SD* = 1.03, 95% CI [2.44, 3.31]; *M*_*retention*_ = 2.57, *SD* = 0.89), 95% CI [2.19, 2.94], post-provocation (*M*_*disposal*_ = 2.49, *SD* = 0.86, 95% CI [2.13, 2.85]; *M*_*retention*_ = 2.51, *SD* = 0.94, 95% CI [2.12, 2.90]), and post-writing (*M*_*disposal*_ = 2.49, *SD* = 0.97, 95% CI [2.08, 2.89]; *M*_*retention*_ = 2.64, *SD* = 1.02, 95% CI [2.21, 3.06]). A 2 × 3 mixed ANOVA revealed that neither the main effects nor interaction were significant (*Fs* < 2.28, *ps* > 0.11, BF_10_s < 0.70).

### Discussion

The results were essentially the same as those of Experiment 1. The disposal group significantly reduced their anger after disposing of the anger-written paper into the shredder. The retention group showed significantly higher anger than the baseline period and disposal group. These results suggest that the results in Experiment 1 could be attributed neither to the physical distance between the participant and the paper nor to the action itself (i.e. embodied cognition). Specifically, Experiment 2 replicated the results of Experiment 1 and excluded the embodied explanation (the sensorimotor experience of throwing the paper) because the action of the disposal group was quite similar to that of the retention group in Experiment 2. The distance between participant and paper was the same in both groups, as the transparent box and shredder were placed on the desk.

### General discussion

This study aimed to determine whether the disposal of anger-written papers could eliminate or at least reduce subjective anger. Disposal manipulation eliminated anger, either by throwing the paper into a trash can or placing it into the shredder. We propose that this anger reduction method is quite effective, so the subjective ratings of anger resumed as much as the baseline levels. We believe that this method can be used in daily life and especially for populations characterised by extreme levels of anger and aggression in their home. The use of this method may potentially contribute to emotion socialization, as parents are the primary model for their children.

These results indicate that the sensorimotor experience of throwing paper plays a small role in reducing subjective anger^44^. Instead, the meaning (interpretation) of disposal plays a critical role. These results are consistent with other studies which showed that the meaning of disposal was critical for determining its impact, not the action itself^30,45^. However, these results are partially inconsistent with those reported by Zhang et al.^44^. Their experiment tested whether certain behaviors could lower the perceived likelihood of bad luck, as is often the case with jinxes. Participants who threw a ball believed that a jinxed-negative outcome was less likely than those who held the ball. They demonstrated that engaging in an avoidant action rather than creating physical distance was critical for reversing the perceived effect of the jinx. The results of Experiment 1 in this study are consistent with their results. However, we demonstrated that neither avoidance action nor physical distance was crucial in reducing subjective anger.

Our results may be related to the phenomenon of ‘backward magical contagion’^47^, which is the belief that actions taken on an object (e.g. hair) associated with an individual can affect the individuals themselves. Rozin et al.^48^ discovered that individuals experience strong negative emotions when their personal objects are possessed by negative others (such as rapists or enemies). However, these emotions are reduced when the objects are destroyed, such as throwing them in a septic tank or burning them. The phenomenon of ‘magical contagion’ or ‘celebrity contagion’ refers to the belief that the ‘essence’ of an individual can be transferred to their possessions. This backward magical contagion operates in a reversed process, where manipulating an object associated with a person is thought to impact the individuals themselves. The current study's findings may be explained by the concept of backward magical contagion, which posits that negative emotions can be transferred from others to an individual through their possessions. This study did not involve the direct mediation of other individuals. The neutralization of subjective anger through the disposal of an object may be achieved by recognizing that the physical entity, such as a piece of paper, has been diminished, thus causing the original emotion to also disappear.

At least, however, some limitations regarding this disposal method should be addressed in future studies. First, the findings of this study are based on the assumption that participants identified their subjective anger with the paper. Thus, subjective anger had gone with the anger-written paper after its disposal. The participants were asked to review the sentences carefully for 30 s to enhance this identification between thought and paper. It is not clear whether this review process is necessary for identification.

Another limitation is that we did not test a digital device, such as a word processor or smartphone, but used only papers. We believe the present disposal method can be generalised to a digital device, whereas empirical data are limited only by physical entities, papers, trash cans, or shredders. Suppose the disposal method is proven to be effective in digital devices. In that case, it will be adopted in various situations, such as business meetings or daily conversations in schools, by writing and disposing of with a smartphone.

Furthermore, although the disposal method had a more significant effect so that the subjective ratings of anger were eliminated as much as the baseline levels, the effectiveness of this method was not directly compared to other anger reduction methods, such as self-distancing. Other methods may be as effective or even more effective than the present disposal method. Personality traits may modulate the effects of anger suppression, although this has not been examined in the techniques used in this or in other studies. Individuals with high (versus low) levels of trait anger tended to experience lapses in effortful control when exposed to anger-relevant stimuli^49,50^. As mentioned above, although cognitive reappraisal (the reinterpretation of the meaning of an unpleasant event) is considered an effective way to reduce anger^12^, it requires more significant cognitive effort^13,14^. Self-distancing is not feasible, particularly during the heat of the moment^13^. Conversely, the disposal method with low cognitive effort used in this study may be more effective for individuals with lower levels of trait self-control than for those with high trait self-control. Future research should examine whether personality traits moderate the relationship between the disposal method and the expected outcomes.

Individuals with higher levels of trait anger tended to have prolonged experiences of induced state anger^51^. However, experimental research on anger regulation strategies has predominantly emphasized the effectiveness of immediate control^10–12^, neglecting to investigate whether these strategies are equally effective in managing anger that persists over time. However, in everyday life, it is not always feasible to implement anger regulation strategies immediately after anger arises. Therefore, to ascertain its practical utility in real-world settings, it is imperative to examine whether the effectiveness of the disposal method varies with the duration of anger.

Moreover, it should be tested whether the disposal method can suppress subjective anger even if participants write down a provocation event in an experiential manner rather than in the analytic rumination manner used in this study. Previous studies suggest that anger rumination can maintain^52^ or even increase^53^ the original level of anger when participants wrote down a provocation event in an experiential rumination manner. As it may not be easy to write down analytically, especially in the heat of the moment, the disposal method will gain further strength if it is valid by experiential rumination.

It should be mentioned that although provocation was effective in both the subjective anger score and the PANAS negative score, the revealed emotion regulation strategy in this study seemed specific to anger (as no significant interaction effect for the PANAS negative score was observed). Kubo et al.^40^ reported that the increase in the state of anger relevant to approach motivation (aggression) by provocation (measured using the STAXI and asymmetry of prefrontal brain activity) was reduced by an apology comment. However, an increase in the subjective scores of negative emotion (assessed using the PANAS) remained unchanged, regardless of the presence or absence of an apology comment. They proposed anger as not a unitary process but one that comprises multiple independent components (subjective anger and negative feelings). If the anger scale used in this study reflects the approach motivation component of anger as well as the STAXI, the disposal method appears to specifically suppress the components of anger’s approach motivation (aggression) and can be used to reduce aggression as a clinical technique.

Despite these limitations, this is the first study to be designed and used to conveniently eliminate subjective anger by interacting with physical entities. It offers a cost-effective and easy-to-use method to reduce anger by rumination about the provocative event, which otherwise lasts longer. Anyone with a pen and piece of paper can use this method. Suppose one maintains a diary or a personal log. In that case, they can write down a provocative event on the day on the memo pad, and throwing it into the trash can eliminate the provocative event. This action may help neutralize the negative emotions associated with the event, potentially protecting the children’s emotional socialization.

## Conclusion

This study presents a new and convenient method for eliminating subjective anger. This method offers a cost-effective way to eliminate anger in various situations, including business meetings, childcare, and clinical applications. The building blocks of this method (e.g. applying it to a digital device or creating a specific application) could be useful in various daily situations as well as behavioural therapies. In particular, for someone who has difficulty suppressing their anger in their homes.

## Data Availability

The datasets used and analysed during the current study available from the corresponding author on reasonable request.
